# Semaphorin 4D produced by human head and neck squamous cell carcinoma induces myeloid derived suppressor cells expansion from peripheral blood monocytes

**DOI:** 10.1186/2051-1426-3-S2-P280

**Published:** 2015-11-04

**Authors:** Rania H Younis, Kyu Lee Han, Tonya Webb

**Affiliations:** 1 School of Dentistry, University of Maryland Baltimore, Baltimore, MD, USA; 2 School of Medicine, University of Maryland Baltimore, Baltimore, MD, USA

## Background

Immune suppression is one of the hallmarks by which head and neck squamous cell carcinoma (HNSCC) develops a tumor permissible environment[1]. Semaphorin 4D (Sema4D), known for its various developmental, physiological and pathological effects, was recently shown to play a role in regulating the immune system[2]. Sema4D is expressed in many epithelial tumors including HNSCC[3]; yet, the effect of Sema4D on modulating the inflammatory profile within the tumor microenvironment of HNSCC remains to be elucidated.

## Methods

Human HNSCC cell lines HN6 and HN13 were used to study the effect of Sema4D produced in tumor conditioned media (TCM), on myeloid and T cells separated from human peripheral blood mononuclear cells (PBMC) of healthy donors. Neutralizing anti-Sema4D antibody and Sema4D immune depletion from the TCM were carried out. Lentivirus shRNA was also used to knock down Sema4D in HNSCC-HN6 cells.

## Results

Treatment of PBMC with TCM from HNSCC HN6 and HN13 cell lines resulted in a significant induction in myeloid derived suppressor cells (MDSC; CD33^+^ CD11b^+^ HLA-DR^-/low^) from freshly isolated CD33^+^ cells. This increase in MDSC corresponded with the suppression of autologous T cell proliferation and a reduction in IFN-γ production. Blockade of Sema4D in the TCM of HN6 and HN13 using anti-Sema4D antibody resulted in a significant reduction in the MDSC population. Furthermore, TCM from HN6 Sema4D-shRNA rescued the MDSC-mediated T cell suppression and recovered IFN-γ, compared to the control. Analysis of the recovered T cell population showed an increase in the effector T cell population (CD4^+^Tbet^+^ and CD8^+^Tbet^+^), and a decrease in regulatory T cells (CD4^+^CD25^+^FoxP3^+^). Mechanistically, we found a decrease in arginase-1 produced by myeloid cells cultured in HN6 Sema4D-shRNA TCM, as well as a reduction in the immune suppressive cytokines, TGF-β, and IL-10.

## Conclusion

This study describes a novel immunosuppressive mechanism for HNSCC through Sema4D induction of MDSCs. Collectively; our work highlights the therapeutic potential of Sema4D inhibition as a strategy to improve the efficacy of immunotherapy in HNSCC.
